# Impact of coxsackievirus-B4E2 combined with a single low dose of streptozotocin on pancreas of outbred mice: investigation of viral load, pathology and inflammation

**DOI:** 10.1038/s41598-019-46227-3

**Published:** 2019-07-12

**Authors:** Mehdi A. Benkahla, Famara Sane, Antoine Bertin, Anais-Camille Vreulx, Firas Elmastour, Hela Jaidane, Rachel Desailloud, Didier Hober

**Affiliations:** 1 Université de Lille, Faculté de Médecine, CHU de Lille, Laboratoire de Virologie/EA3610, F-59037 Lille, France; 2 Université de Monastir, Faculté de Pharmacie, Laboratoire des Maladies Transmissibles et Substances Biologiquement Actives LR99ES27, Monastir, Tunisia; 3 Université de Tunis El Manar, Faculté des Sciences de Tunis, Tunis, Tunisia; 4 Université de Picardie Jules Verne, CHU d’Amiens, Service d’Endocrinologie-Diabétologie-Nutrition, F-80054 Amiens, France

**Keywords:** Viral pathogenesis, Virus-host interactions

## Abstract

Coxsackieviruses B (CV-B) belong to the EV-B species. CV-B and particularly CV-B4 are thought to be involved in the development of chronic diseases like type 1 diabetes (T1D). The mechanisms of the enteroviral pathogenesis of T1D are not well known, yet. The *in vitro* studies are rich with information but *in vivo* infection models are needed to investigate the impact of viruses onto organs. Our objective was to study the impact of CV-B4E2 combined with a single sub-diabetogenic dose of streptozotocin (STZ) on the pancreas of mice. The infection with CV-B4E2 of CD1 outbred mice treated with a sub-diabetogenic dose of STZ induced hyperglycemia and hypoinsulinemia. Along with the chemokine IP-10, viral RNA and infectious particles were detected in the pancreas. The pancreas of these animals was also marked with insulitis and other histological alterations. The model combining STZ and CV-B4E2 opens the door to new perspectives to better understand the interactions between virus and host, and the role of environmental factors capable, like STZ, to predispose the host to the diabetogenic effects of enteroviruses.

## Introduction

Coxsackievirus B (CVB) 1 to 6, are viruses with RNA genome that belong to the species *Enterovirus B* (EV-B), of the *Enterovirus* genus of the *Picornaviridae* family. CVB causes asymptomatic infections in humans but also various acute diseases like pancreatitis, myocarditis and aseptic meningitis, as well as chronic diseases such as dilated cardiomyopathy^1,2^ and type 1 diabetes (T1D)^1–4^. T1D is a proinflammatory progressive disease characterized by specific islet β cell destruction or dysfunction and loss of insulin secretion. The role of CV-B, especially CV-B4, in the pathogenesis of T1D is strongly suspected^3–5^.

During CV-B4 infection, the viral RNA is detected by TLR3^6^, which activates the Toll/interleukin-1 receptor domain-containing adaptors inducing IFN (TRIF) that then induces the expression of type I IFNs and the overexpression of IFN-inducible protein 10 (IP-10). TLR3 also activates Nuclear Factor Kappa Beta (NF-κβ) which stimulates the production of pro-inflammatory cytokines such as IL-1β, IL-6 and TNFα^7–9^. Several studies have reported that lymphocytes infiltrating islets overexpress IP-10 and that β cells under the influence of cytokines (TNFα, IFNγ) produce IP-10. Furthermore, IP-10 has been identified as the most dominant chemokine expressed in the islet’s environment of prediabetic animals and T1D patients^10–14^.

Type 1 diabetes can be induced in animals by various means^15–17^. Diabetes can be induced by streptozotocin (STZ), a glucosamine-nitrosourea compound that specifically targets pancreatic β cells. It was isolated for the first time from the *Streptomyces achromogenes* bacteria. The molecular structure is a 2-deoxy-D-glucose molecule substituted by an N-methyl-N-nitrosourea group at the carbon C2 level^18^. The selective toxicity towards pancreatic β cells is related to the glucose fraction in its chemical structure, which allows the STZ to enter the cell via the low affinity glucose transporter-2 (GLUT2). The β cells are also more sensitive to STZ because they are more active than other cells to incorporate glucose^19^. Insulin-producing cells that do not express GLUT2 are resistant to STZ toxicity^19^. Once inside, it is rapidly metabolized and specifically affects the DNA and the mitochondria. In mitochondria, STZ acts by inhibiting the production of ATP and by affecting NAD (Nicotinamide Adenine Dinucleotide) at the same time^20^. Furthermore, STZ binds to DNA to form dimers (DNA adducts), activating the DNA repair process that uses a poly (ADP-ribose) polymerase converting NAD to ADP-ribose^21^. Since the production of NAD is disrupted, the enzyme cannot perform its function, which leads to the death of insulin secretory cells by apoptosis or necrosis, which contributes to the development of diabetes. The administration of multiple low doses of STZ in animals induces initial cell damage by alkylating the DNA in islet cells and/or by spontaneously releasing NO. Then, mononuclear cells infiltrate the islets which triggers a process that results in islet destruction and diabetes^22–24^. It was reported that chemokines, like IP-10, are critical determinants of multiple low doses of STZ-induced diabetes^13^.

It was reported that inbred mice treated with a single sub-diabetogenic dose of streptozotocin developed diabetes when they were infected with EMC virus, CV-B3 or CV-B5^25^. The CD1 mouse strain is derived from a Swiss stock and is of great interest for toxicology and virology since its genetic structure is close to that of natural Mus musculus populations. CD1 mice are susceptible to diabetogenic viruses such as EMCV and CV-B4^26^. The purpose of the present study was to investigate the impact of CV-B4E2 combined with a single sub-diabetogenic dose of streptozotocin on the pancreas and on pancreatic β cells of outbred mice.

## Results

### Diabetes induced by CV-B4E2 in mice treated with a sub-diabetogenic dose of streptozotocin

Streptozotocin solubilized in sodium citrate buffer (pH 4.5) was injected intraperitoneally at various concentrations to 3 week old male CD1 mice. It was determined that the sub-diabetogenic dose of STZ not causing hyperglycemia was 35 mg/kg while the diabetogenic dose was 45 mg/kg and more (Supplementary Fig. S1).

**Figure 1:**
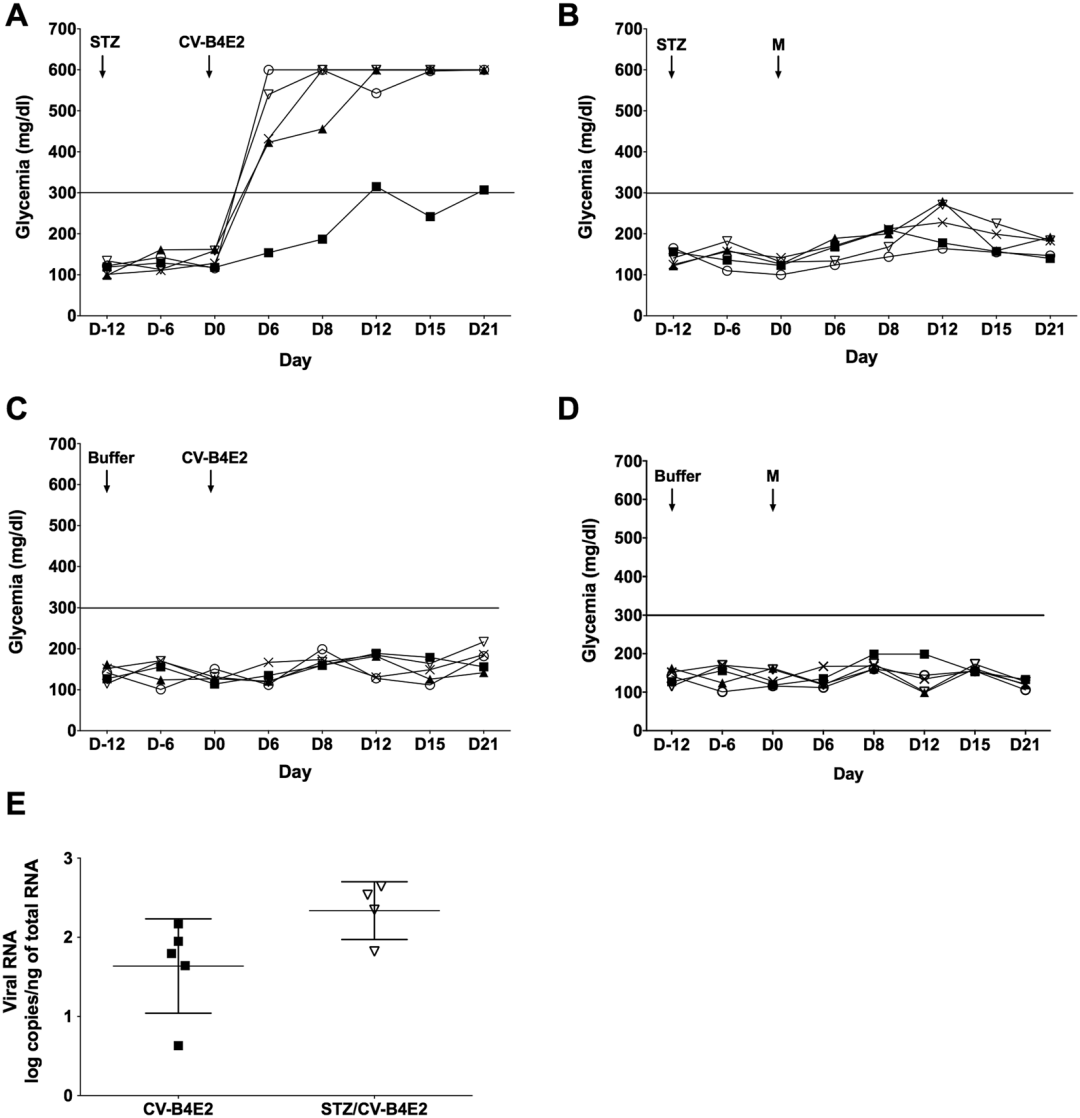
Individual monitoring of blood glucose levels and quantification of enteroviral RNA in mouse pancreas. CD1 mice were intraperitoneally (ip) injected with 35 mg of STZ/kg (**A**,**B**) or buffer (Buffer) (**C**,**D**) and 12 days later with CV-B4E2 (2.10^4^ TCID_50_) or culture medium (M) using the same route. A drop of blood was collected from the tail vein regularly from the day of injection of STZ or buffer (Buffer) until 21 days after inoculation with CV-B4E2 or medium. The blood glucose level is measured using a glucometer. The results are expressed as mg/dl. 21 days post-infection, the mice were sacrificed by cervical dislocation and the pancreas was removed to investigate the presence of viral RNA by quantitative RT-PCR. The results are mean +/− SD of log copies per ng of total RNA. Each mouse is represented by a symbol, with five mice in each group. 300 mg/dl is the threshold above which mice are considered hyperglycemic.

Mice pretreated with 35 mg of STZ/kg or sodium citrate buffer were inoculated 12 days later with CV-B4E2 (2.10^4^ TCID_50_) or culture medium (Fig. 1).

Blood glucose levels in control mice injected with culture medium, CV-B4E2, or a sub-diabetogenic dose of STZ were lower than 300 mg/dl (300 mg/dL is the threshold for hyperglycemia) whereas they were higher in animals treated with STZ combined with CV-B4E2. Blood glucose values ranging from 423 to 600 mg/dL and above (600 mg/dL was the upper limit of detection of the test) were measured day 6 through day 21 after virus inoculation in 4 out of 5 mice. The blood glucose levels day 6 through day 21 were higher than those of controls (p < 0.05). In one event, one mouse had a blood glucose level of 300 mg/dl 12 days post-inoculation (see Fig. 1A–D).

Twenty days after inoculation, the mice were sacrificed and the pancreas and blood were recovered. The detection of infectious particles by end-point dilution assay in pancreatic tissue homogenates was negative (data not shown). In contrast, there was enteroviral RNA in the pancreas of CV-B4E2-infected mice regardless of whether it was treated with STZ: 2.33 +/− 0.36 and 1.63 +/− 0.59 log copies/ng of total RNA respectively (p > 0.05) (Fig. 1E).

Twelve days after being treated with sub-diabetogenic dose of STZ (35 mg/kg), the three week old male CD1 mice were infected with CV-B4E2 (2.10^4^ TCID_50_). The mice were sacrificed on day 5, 10, 15, 20, and 25 post-infection to recover the pancreas and blood; their blood glucose levels were also measured on the days they were sacrificed.

The blood that was obtained by cardiac puncture from euthanised mice was centrifuged and the serum recovered to measure the level of insulin by ELISA.

**Figure 2:**
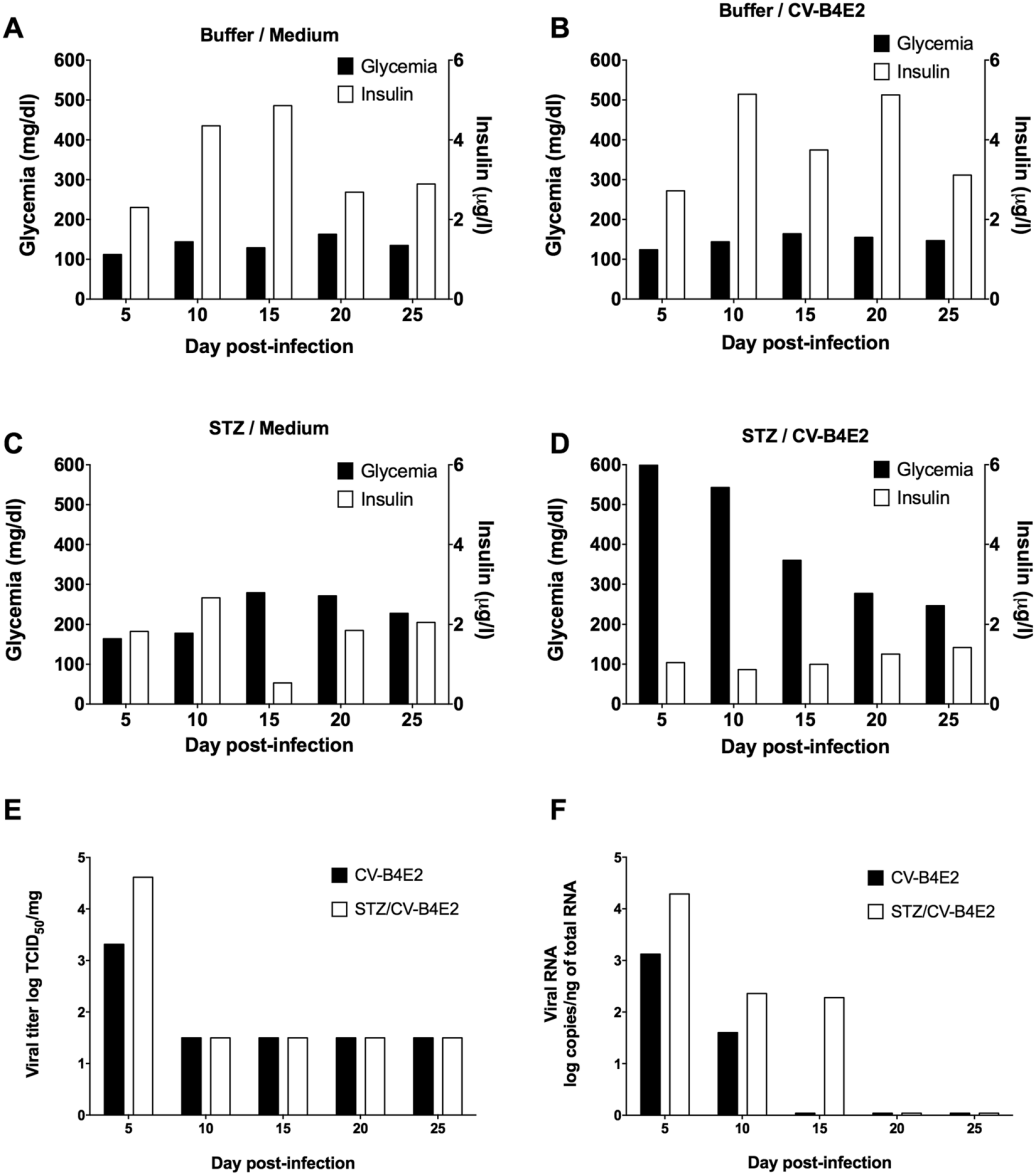
Diabetes induced by CV-B4E2 in mice treated with a sub-diabetogenic dose of STZ. Mice that received STZ or buffer, and then inoculated 12 days later with CV-B4E2 or culture medium, were sacrificed on day 5, 10, 15, 20 and 25 post-infection (one mouse at each time point to collect blood and pancreas). Blood glucose and insulin levels were measured (**A**–**D**) and the pancreas was recovered to investigate the presence of infectious particles by endpoint dilution assay; the results are expressed as TCID_50_/mg (**E**). The method used to investigate the presence of viral RNA was by quantitative RT-PCR; the results are expressed as log of viral RNA copies by ng of total RNA (**F**).

The blood glucose levels in mice inoculated with culture medium or with CV-B4E2 ranged between 112 and 164 mg/dl and the serum insulin levels were 2.30 to 5.14 μg/l (Fig. 2A,B). In STZ-treated mice, the blood glucose levels at day 15, day 20 and day 25 were slightly higher (up to 279 mg/dl) and the blood insulin levels ranged between 0.53 and 2.66 μg/l (Fig. 2C). In mice inoculated with STZ and then infected with CV-B4E2 12 days later (Fig. 2D) the levels of blood glucose were higher compared with the values obtained in controls (p < 0.05) (Fig. 2A–C). In mice inoculated with STZ and CV-B4E2 the levels of insulin were lower than those of animals inoculated with medium or CV-B4E2 (p < 0.05), and to a lesser extent than those of animals inoculated with STZ (p > 0.05) (Fig. 2A–D)

Infectious particles were found on day 5 in the pancreas of mice infected with CV-B4E2 regardless of whether they were treated with STZ. On day 10, day 15, day 20 and day 25, the detection of infectious particles in the pancreas of both groups of mice was negative (Fig. 2E).

Enteroviral RNA was found in the pancreas recovered from mice inoculated with CV-B4E2 on day 5 and 10 post-infection and from mice inoculated with STZ/CV-B4E2 on day 5, 10 and 15 post-infection. In pancreas harvested after 15 days post-infection, viral RNA was undetectable in mice infected with CV-B4E2 regardless of whether they were treated with STZ (Fig. 2F).

### Impact of CV-B4E2 on the pancreas of mice treated with a sub-diabetogenic dose of streptozotocin

In mice inoculated with a sub-diabetogenic dose of STZ and CV-B4E2, hyperglycemia and hypoinsulinemia were observed on day 5 post-infection. Therefore it was decided to use this model to study viral load and inflammatory cytokines in pancreas collected on day 5 post-infection and to analyze the impact of the virus through a histological analysis of the pancreas.

**Figure 3:**
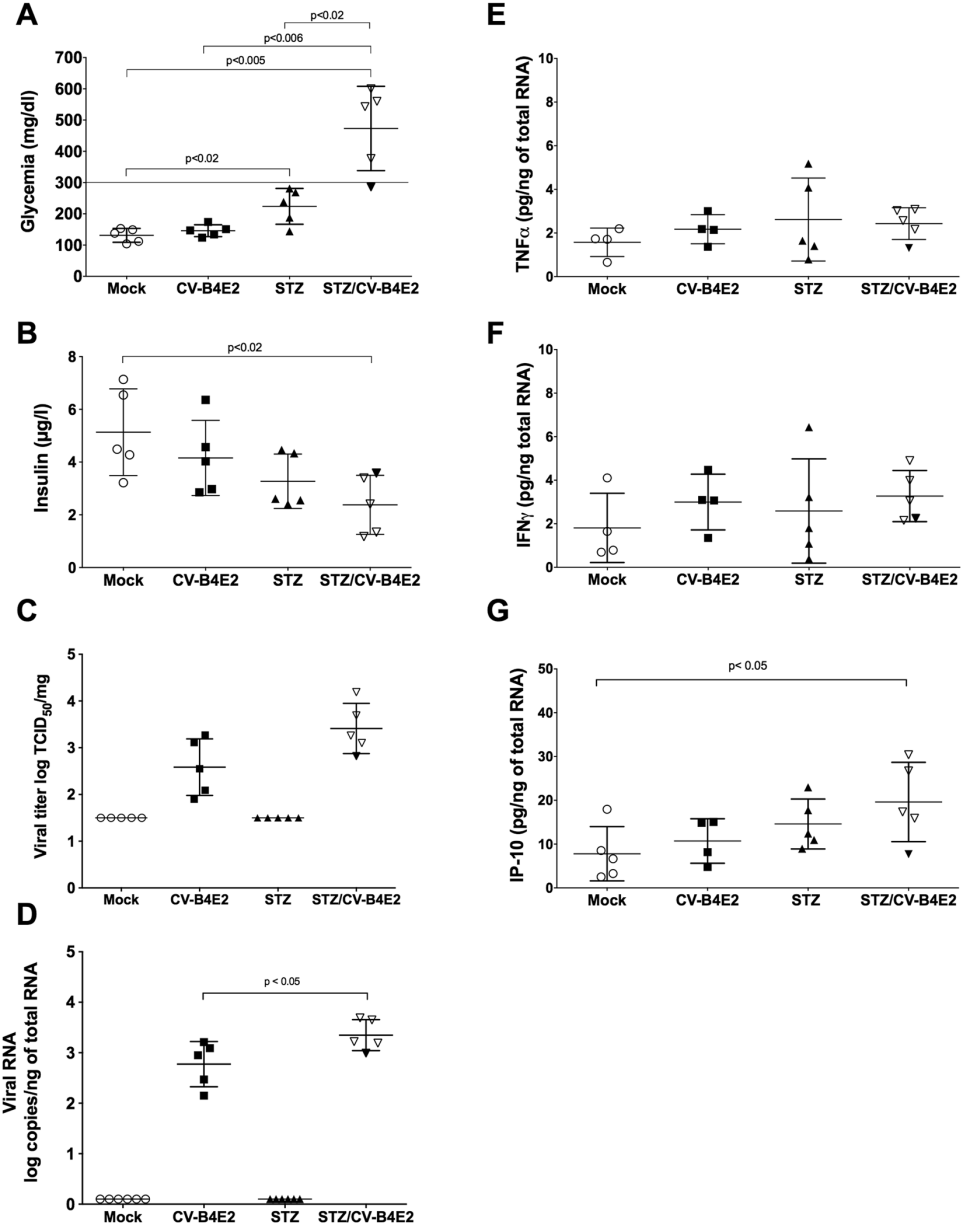
Pattern of inflammatory cytokines in pancreas of CD1 mice. CD1 mice were treated with STZ (35 mg/kg) and then 12 days later were inoculated with CV-B4E2 (2.10^4^ TCID_50_) (STZ/CV-B4E2). Controls were treated with buffer or STZ and then inoculated with culture medium (Mock and STZ respectively) or with CV-B4E2 (CV-B4E2). On day 5 post-infection blood was collected from the tail vein to determine the level of blood glucose by using a glucometer; (**A**) then the animals were euthanised and the blood obtained by cardiac puncture was centrifuged and the serum recovered to measure the level of insulin by ELISA (**B**). The pancreas was harvested in presence of protease inhibitors and processed as described in the materials and methods section to determine the level of infectious particles (**C**) and of enteroviral RNA (**D**) – expressed as log TCID_50_/mg of organ and as log of the number of copies of enteroviral RNA/ng of total RNA, respectively. TNFα (**E**), IFNγ (**F**) and IP-10 (**G**) in pancreas were quantified by ELISA, the results were expressed as pg per ng of total RNA. The results are mean +/− standard deviation (5 mice in each group). The dark inverted triangle in the group STZ/CV-B4E2 represents the animal with blood glucose level < 300 mg/dl.

In mice treated with STZ and then infected with CV-B4E2, the levels of blood glucose were higher and the levels of blood insulin were lower compared with controls on day 5 post inoculation or mock-infected. 4 out of 5 mice had a blood glucose level above 300 mg/dl (see Fig. 3A). In mice treated with STZ, the levels of blood glucose were increased as well but at a lesser extent (224 +/− 57.37 mg/dl). Infectious particles were found in the pancreas of CV-B4E2-infected mice regardless of whether they were treated with STZ (3.41 +/− 0.53 vs 2.58 +/− 0.60 log TCID_50_/mg p > 0.05). Enteroviral RNA was found in the pancreas of CV-B4E2-infected mice and STZ/CV-B4E2-infected mice (2.77 +/− 0.44 vs 3.34 +/− 0.30 log copies/ng of total RNA, p < 0.05 (Fig. 3D).

In CV-B4E2-infected mice treated with STZ, the mean levels of TNFα and IFNγ in pancreatic tissues were not higher than those obtained in pancreas of mice inoculated with buffer and medium (mock) (see Fig. 3E,F) whereas the mean levels of IP-10 were higher (Fig. 3G) (19.7 +/− 8.9 vs 7.8 +/− 6.2 pg/ng of total RNA p < 0.05). It was noted that the level of IP-10 was low (7.70 pg/ng of total RNA) in the animal with blood glucose lower than 300 mg/dl (see the dark inverted triangle symbol in Fig. 3G).

When mice treated with STZ were inoculated wih UV-inactivated CV-B4E2, the blood glucose levels were not higher than those of controls (ranging values: 112 to 168 mg/dl) (data not shown).

**Figure 4:**
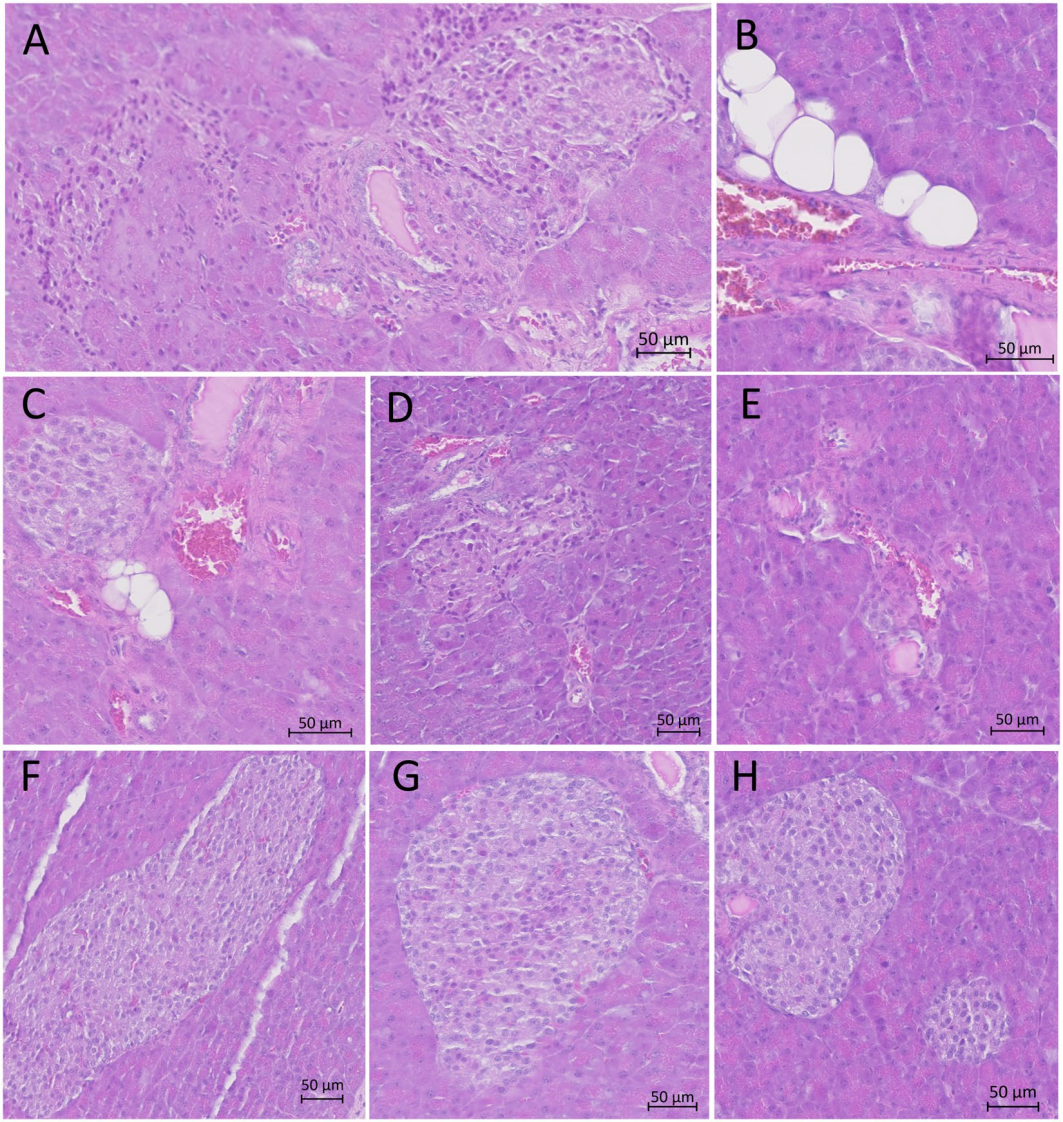
Pancreatic lesions in mice treated with streptozotocin and inoculated with CV-B4E2. CD1 mice were treated with STZ (35 mg/kg) and then 12 days later were inoculated with CV-B4E2 (2.10^4^ TCID_50_). Controls were treated with buffer or STZ then inoculated with culture medium or with CV-B4E2. The animals were sacrificed at day 5 post inoculation. The pancreas was collected in buffered formalin and then processed as described in the Materials and Methods section. The pictures show a representative aspect of lesions of pancreas from mice treated with STZ prior to inoculation with CV-B4E2: inflammatory infiltrate (**A**), fat involution (**B**,**C**) and abnormal morphology of endocrine tissue (**D**,**E**). They also show a representative aspect of pancreas from controls treated with buffer (**F**) or STZ (**G**) then inoculated with culture medium (**F**,**G**) and a representative aspect of pancreas from controls treated with CV-B4E2 (**H**).

The histological analysis of the pancreas of 5 out of 6 CV-B4E2-infected mice treated with STZ, with blood glucose levels >300 mg/dl, displayed an inflammatory infiltrate of exocrine tissue and around Langerhans islets, an adipose involution and abnormal morphology of endocrine tissue. In contrast, there was no lesion of pancreas obtained from the CV-B4E2-infected mice treated with STZ, of which the blood glucose level was <300 mg/dl and from control mice that were inoculated with buffer and culture medium, STZ or CV-B4E2 (Fig. 4).

## Discussion

This study is different in many aspects from those of other investigators who reported that inbred mice exposed to a sub-diabetogenic dose of STZ followed by inoculation of an enterovirus (CV-B3 or CV-B5) or a cardiovirus (EMC) developed hyperglycemia^25^. In the present study, the diabetogenic effect of CV-B4E2 inoculated in outbred CD1 mice treated with STZ was observed, and the relationship between hyperglycemia and hypoinsulinemia as well as the presence of viruses and inflammatory markers in the pancreas was investigated.

Several considerations in the present report are noteworthy. Blood glucose level higher than 300 mg/dl was obtained in mice receiving a single non-diabetogenic dose of STZ (35 mg/kg) by the intraperitoneal route prior to the inoculation of CV-B4E2 by the same route (2.10^4^ TCID_50_) 12 days later. In contrast, when mice received either the same dose of STZ or the same viral inoculum, the blood glucose levels remained below 300 mg/dl. The blood glucose values observed in non-fasting CD1 mice in our experiments (100–150 mg/dl) were close to those measured in non-fasting SJL mice (80–120 mg/dl) by other investigators^27^. The infection can only be confirmed by the presence of virus in tissues. The infection of mice in our study was evidenced by the presence of infectious viral particles and by the quantification of viral RNA in pancreas. Infectious viral particles were found in the organ 5 days after inoculation of CV-B4E2 but not later than that. Nevertheless, viral RNA was detected upto 15 days post-inoculation but not on day 20 and beyond in most animals. In mice sacrificed on day 20 and day 25, enteroviral RNA was not found suggesting that the virus had been cleared. In some STZ-treated mice inoculated with CV-B4E2, normal blood glucose and insulin levels indicated that the virus had no impact on glucose homeostasis – possibly because of a low inoculum. In contrast, it was observed that in a STZ-treated mouse infected with the virus, enteroviral RNA was present in the pancreas on day 21. Nevertheless, blood glucose and insulin levels were normal, suggesting that the diabetogenic effect of CV-B4E2 in animals exposed to STZ depended on host factors. Whatever these factors, clearly the effect of the virus in mice treated with STZ depended on the occurrence of an infection since animals receiving STZ followed by inactivated CV-B4E2 did not develop hyperglycemia.

The role of inflammation in the development of type 1 diabetes is strongly suspected^28^. In mice treated with STZ prior to the infection with CV-B4E2, the inflammation of pancreas was studied. Disparities concerning glucose homeostasis as well as the presence of virus in pancreas were observed 5 days after the inoculation of CV-B4E2; therefore, the inflammatory profile was investigated in the organ collected on that date. The pancreas was prepared in presence of protease inhibitors and inflammatory cytokines (IP-10, TNFα and IFNγ). The concentrations were determined using enzyme immunoassays, as described previously, to study the inflammatory profile of various tissues in animals exposed to LPS^29^. In the present study, the results were standardized with respect to the total amount of RNA contained in the organ fragment instead of the total amount of protein as described by other researchers^29^. Thus it was observed that the pancreatic concentrations of TNFα and IFNγ were not significantly different in control mice and in animals exposed to either STZ or CV-B4E2 or to a combination of both. In contrast, the pancreatic levels of IP-10 in hyperglycemic mice exposed to STZ prior to CV-B4E2 were significantly higher than in controls. Nevertheless, the values were not higher than those obtained in animals which received either STZ or CV-B4E2 and that were not hyperglycemic. Thus, the increase in the concentration of pancreatic IP-10 was not related to the hyperglycemia observed in mice treated with STZ prior to CV-B4E2 inoculation. However, it was observed that IP-10 values were higher in animals with hyperglycemia than in the others without hyperglycemia. All these animals were infected as evidenced by the detection of infectious virus particles and viral RNA in their pancreas. It can be assumed that the infection of pancreatic cells was involved in the impact of CV-B4 infection onto STZ-treated mice. It had already been reported that CBV-4 could replicate and induce CPE in mouse pancreatic β cells *in vitro*^30^. It was also observed that pancreatic sections from mice inoculated with CV-B4 showed the localization of the viral protein VP1 within insulin+ β cells and within exocrine tissue^31^. Furthermore, it was demonstrated that CVB can replicate in islets of mice when the intraislet environment is suitably altered^32^. It can be hypothesized that a direct effect of CV-B4 on β cells and/or other pancreatic cells resulted in hyperglycemia and pancreatic tissue lesions observed in STZ-treated animals inoculated with the virus. Indeed, the levels of infectious virus and viral RNA were around 4 log TCID50/mg of tissue and 4 log viral RNA copies/ng of total RNA respectively. However, if the proportion of infected β cells was low, an indirect effect through the release of various mediators cannot be excluded. Further studies are needed to determine the relationship between the extent of infection of β cells by the virus and its impact onto the pancreas in mice treated with a single subdiabetogenic dose of STZ.

The levels of IP-10, but not those of TNFα and IFNγ, increased in mice exposed to STZ and CV-B4E2; therefore, it can be suggested that the inflammatory response was more likely related to pancreatic cells producing IP-10 rather than immune system cells producing TNFα and IFNγ – otherwise all the cytokine levels would have increased^10,33^. This pattern of inflammatory reaction was also observed in mice exposed to STZ or CV-B4E2, but under these conditions the animals were not hyperglycemic. Thus, the excessive levels of IP-10 were not correlated with the hyperglycemia induced in mice by the combined effect of STZ and CV-B4E2. Nevertheless, in the pancreas of each hyperglycemic mouse treated with STZ prior to inoculation with CV-B4E2, lesions and inflammatory infiltrates of endocrine tissue were observed, whereas there was no lesion in the pancreas from a normoglycemic mouse exposed to the same treatment as well as from normoglycemic mice exposed to STZ or CV-B4E2. Altogether, these results indicate that in STZ-treated mice, CV-B4E2 can induce alterations in endocrine pancreatic tissue that are associated with disparities of glucose homeostasis observed in these animals. It remains to be determined whether other cytokines than IP-10 and/or other factors induced by CV-B4E2 in mice treated with STZ are involved in hypoinsulinemia associated with hyperglycemia.

Hypoinsulinemia in animals was associated with lesions of pancreatic tissue, which was probably due to direct and indirect effects of the virus, including inflammation response of the tissue, especially production of IP-10, and intervention of other factors. Interestingly, it has been reported that STZ in rat β pancreatic cells (cell line INS1) negatively regulated the PI3k/AKT/ERK/mTOR pathway and had a significant impact on β cell survival, normal morphology, cell mass, and reduced intracellular insulin production. This pathway was involved in CV-B-induced autophagy in HeLa cells as well^34,35^. Whether or not a subdiabetogenic dose of STZ combined with CV-B4E2 has an impact on the PI3k/AKT/ERK/mTOR pathway resulting in a disturbance of glucose homeostasis *in vivo* is an open issue. The mechanisms of hypoinsulinemia in mice treated with STZ and then inoculated with CV-B4E2 remains to be elucidated.

The rapid onset of diabetes induced by STZ, prior to inoculating CV-B4E2, in mice in our experiments is reminiscent of a similar pattern of disturbance of glucose homeostasis observed in humans with fulminant diabetes characterized by the rapid onset of hyperglycemia resulting from a failure/accelerated alteration of pancreatic islet cells^36,37^. The model combining STZ and CV-B4E2 in CD1 mice opens the door to new perspectives to better understand the mechanisms of fulminant diabetes. Moreover, in the context of the hypothesis of the role of enteroviruses in autoimmune type 1 diabetes, which is more common compared to fulminant diabetes, the model combining STZ and CV-B4E2 on one hand opens perspectives to better understand the interactions between virus and host that participate in the pathogenesis of enterovirus-induced disease, and on the other hand opens perspectives to study the role of environmental factors capable, like STZ, to predispose the host to the diabetogenic effects of enteroviruses. Future studies will be directed along this line in the laboratory.

## Material and Methods

### Streptozotocin

Streptozotocin (Sigma-Aldrich, St-Louis, MO, USA) was dissolved in sodium citrate buffer (Sigma-Aldrich, pH 4.5) at a concentration of 22.5 mg/ml. A dose of 35 mg/kg was immediately injected intraperitoneally. The STZ solution must be extemporaneously prepared^38^.

### Cell lines

HEp-2 cells (ATCC, Manassas, VA, USA) cultured in MEM medium (Thermofisher Scientific, Waltham, MA, USA) supplemented with 10% fetal bovine serum (FBS), 1% L-glutamine, 50 μg/ml of streptomycin, 50 UI/ml of penicillin (BioWhittaker, Walkersville, MD, USA) and 1% non-essential amino acids (Thermofisher Scientific) at 37 °C and incubated in a 5% CO2 atmosphere.

### Virus, virus titraiton and viral inactivation by UV

CV-B4E2 provided by Ji-Won Yoon (Calgary, Canada) was propagated on HEp-2 cells (ATCC). Three days post inoculation, the flasks are freeze-thawed three times, and centrifuged at 2,000 × g for 10 min at 4 °C. The supernatant was aliquoted and stored at −80 °C.

The virus titers in supernatants of cell cultures or organ homogenates were assessed using the end-point dilution assay and the Spearman-Karber method was used to calculate the tissue culture 50% infectious dose (TCID50)^39^.

For UV inactivation, CV-B4E2 was diluted in MEM medium and subsequently irradiated in six-well cell culture dish for 10 minutes at an intensity of 254 µW/CM2 using a Germicidal UV lamps. Type: VL-4C (Vilber, Germany).

### Mice

CD1 male mice were obtained from Envigo RMS Sarl (Harlan Laboratories, Gannat, France). All experiments were done in accordance with French and European guidelines for animal care, and were approved by the Ethical committee for animal experimentation of Nord-Pas-de-Calais (France). Mice were given ad libitum access to sterile water and commercial sterile food pellets (SAFE, Augy, France) and were maintained in a 12 h light/dark cycle. All blood glucose measurements were done in the morning, the mouse was placed in a tube restrainer then the tail vein was punctured with a 30G needle and a small droplet of blood was placed into the blood glucose strip inserted in the glucometer (Bayer Healthcare, Loos, France).

CD1 male mice, aged 3 weeks, were inoculated intraperitoneally with a dose of 35 mg STZ/kg. At day 12, following treatment with STZ, these same mice were inoculated with 200 μl of a suspension of CV-B4E2 at 2.10^4^ TCID_50_. At the end of the follow-up, the animals were sacrificed by cervical dislocation, and their pancreas is recovered in buffered formalin for histopathology or frozen at −80 °C for RNA extraction or infectious particles titration.

### RNA extraction

The pancreas is first weighed and then crushed using a tissue ruptor (Qiagen, Hilden, Germany) in 1 ml of PBS, supplemented with a protease inhibitor cocktail (ULTRA Tablets, Mini, EASYpack, Roche, Basel, Switzerland) and then centrifuged at 2,500 × g at 4 °C for 10 min and then aliquoted and stored at −80 °C.

Total RNA was extracted using Tri-Reagent (Sigma-Aldrich), as described elsewhere^40^. The RNA thus obtained is aliquoted and stored at −80 °C until use. One of the aliquots is directly used for the RNA quantification by Nanodrop spectrophotometer (Thermofisher Scientific).

### Quantitative RT-PCR

The RNA retrotranscription was performed using the Affinityscript® QPCR cDNA Synthesis kit (Agilent technologies, Les Ulis, France). Quantitative real-time PCR for cDNA amplification was carried out with the Brilliant® II QPCR kit (Agilent technologies) on the Mx3000p® instrument (Agilent technologies). Enterovirus 71 RNA (Vircell, Granada, Spain) was used as standard for quantification. The oligonucleotides and the reaction conditions were described previously^41^.

### Measurement of IP-10, TNFα, IFNγ and Insulin protein

Fragments of pancreas were homogenized in 1 ml of PBS supplemented with a protease inhibitor cocktail (cOmplete ULTRA Tablets, Mini, EASYpack, Roche) after centrifugation at 2500 g at 4 °C for 10 min. The supernatant was harvested and stored at −80 °C. The supernatant was used to evaluate the levels of IP-10, TNFα and IFNγ. The results were normalized with total RNA harvested from cells.

IP-10, TNFα (Thermofisher Scientific), IFNγ (Covalab, Villeurbanne, France) were measured by ELISA according to the manufacturer’s instructions. The sensitivity of each assay was 7.8 pg/ml, 15 pg/ml, and 15 pg/ml respectively.

Blood was obtained by cardiac puncture from euthanised mice to measure the level of insulin by ELISA. according to the manufacturer’s instructions, the sensitivity of the assay was 0.2 μg/l (Mercodia, Uppsala, Sweden).

### Histological analysis

The organs were fixed in buffered formalin, embedded in paraffin, and cut into 5 μm thick sections. The sections are subsequently deparaffinized and then dehydrated with xylene for 10 minutes. The cuts are then rehydrated by decreasing degrees of ethanol (100, 95, 70 and 50°) and finally with double distilled water. Staining is done for 15 min in hematoxylin, followed by washing with running water, a differentiation step which consists of a 1% HCl ethanol bath, and then incubation in water for 5 min. Staining of the cytoplasm is carried in eosin for 5 min followed by washing with water to remove excess dye. The sections are again dehydrated and mounted on superfrost plus slides for observation using Axioscan (Z1) scanner (Zeiss, Oberkochen, Germany). The histological examinations were blinded.

### Statistical analysis

The statistical analysis of the results was performed with the student T-test and Welch’s correction test, when appropriate (GraphPad Prism quickCalcs, San Diego, CA, USA). The differences were considered significant for a p value < 0.05.

## Supplementary information

Supp figure 1
