# Supplementary material for: Impact of coxsackievirus-B4E2 combined with a single low dose of streptozotocin on pancreas of outbred mice: investigation of viral load, pathology and inflammation
Source: Sci Rep. 2019 Jul 12;9:10080. doi: 10.1038/s41598-019-46227-3 (PMC6626040; doi:10.1038/s41598-019-46227-3)
Supplement: Supplementary file 1 — Supp figure 1 [file 41598_2019_46227_MOESM1_ESM.pdf]

Impact of coxsackievirus-B4E2 combined with a single low dose of streptozotocin on pancreas of outbred mice: investigation of viral load, pathology and inflammation

Mehdi A. Benkahla[a], Famara Sane[a], Antoine Bertin[a], Anais-Camille Vreulx[a], Firas Elmastour[a], Hela Jaidane[b,c], Rachel Desailloud[d], Didier Hober[a]

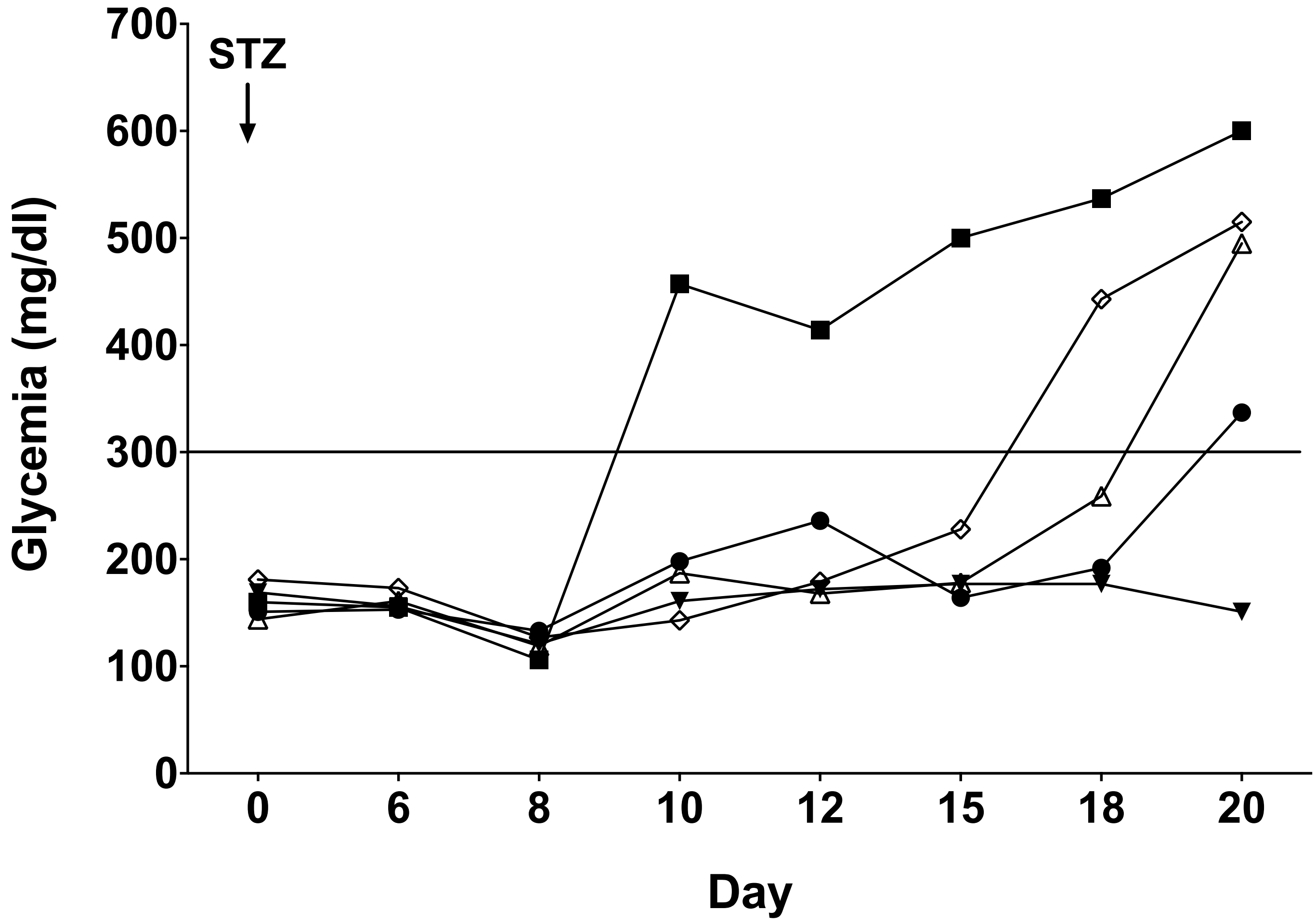

Supplementary figure S1 : Streptozotocin-induced hyperglycemia in CD1 mice. CD1 mice were inoculated with 45 mg of STZ/Kg intraperitoneally**.** Each mouse is represented by a symbol (n=5). Blood was collected from tail vein until day 20 post-infection. The blood glucose level was measured using a glucometer. The results are expressed as mg/dl. 300 mg/dl is the threshold for hyperglycemia
